# Serum Creatinine Levels and Nephrocheck® Values With and Without Correction for Urine Dilution-A Multicenter Observational Study

**DOI:** 10.3389/fmed.2022.847129

**Published:** 2022-02-18

**Authors:** Robert G. Hahn, Fumitaka Yanase, Joachim H. Zdolsek, Shervin H. Tosif, Rinaldo Bellomo, Laurence Weinberg

**Affiliations:** ^1^Karolinska Institute at Danderyd's Hospital (KIDS), Stockholm, Sweden; ^2^Department of Research, Sodertalje Hospital, Sodertalje, Sweden; ^3^Australian and New Zealand Intensive Care Research Centre (ANZIC-RC), School of Public Health and Preventive Medicine, Monash University, Melbourne, VIC, Australia; ^4^Department of Biomedical and Clinical Sciences (BKV), Linköping University, Linköping, Sweden; ^5^Department of Anaesthesia, Austin Hospital, Melbourne, VIC, Australia; ^6^Department of Critical Care, The University of Melbourne, Melbourne, VIC, Australia

**Keywords:** urine, dilution addition, acute kidney disease, surgery, anesthesia

## Abstract

**Background:**

The Nephrocheck® test is a single-use cartridge designed to measure the concentrations of two novel cell-cycle arrest biomarkers of acute kidney injury, namely tissue inhibitor of metalloproteinase 2 (TIMP-2) and insulin-like growth factor binding protein 7 (IGFBP7). Correlations of serum creatine values and TIMP-2 and IGFBP7 with and without correction for urine dilution have not been previously undertaken in patients undergoing major abdominal surgery. We hypothesized that the Nephrocheck® values would be significantly different with and without correction for urine dilution in patients with elevated creatinine values post major abdominal surgery.

**Methods:**

We performed a *post hoc* analysis of serum and urine specimens sampled preoperatively and postoperatively in 72 patients undergoing major abdominal surgery. Thirty samples were measured from patients with the greatest decrease and the greatest increase in postoperative serum creatinine values. Urine was analyzed with the Nephrocheck to predict the risk of acute kidney injury (AKIRisk™). We then examined the relationship between serum creatinine and the urinary excretion of TIMP-2 and IGFBP7 as measured by the Nephrocheck test. The AKIRisk between the groups with and without correction for urine dilution was assessed.

**Results:**

The median perioperative change in serum creatinine in the two groups was −19% and +57%, respectively. The uncorrected median baseline AKIRisk decreased from 0.70 (25th−75th percentiles, 0.09–1.98) to 0.35 (0.19–0.57) (mg/L)^2^ in the first group and rose from 0.57 (0.22–1.53) to 0.85 (0.67–2.20) (mg/L)^2^ in the second group. However, when corrected for the squared urine dilution, the AKIRisk™ in patients with postoperative increases in serum creatinine was not indicative of kidney injury; the corrected AKIRisk was 8.0 (3.2–11.7) μg^2^/mmol^2^ before surgery vs.6.9 (5.3–11.0) μg^2^/mmol^2^ after the surgery (*P* = 0.69).

**Conclusion:**

In the setting of major abdominal surgery, after correction of TIMP-2 and IGFBP7 for urine dilution, the Nephrocheck AKIRisk scores were significantly different from the uncorrected values. These finding imply that the AKIRisk index is a function of urine flow in addition to an increased release of the biomarkers.

## Introduction

Serum creatinine is the key biomarker used to diagnose acute kidney injury (AKI) after major surgery. Besides low urine output, the consensus criteria for Stage 1 AKI is an increase of serum creatinine by >50% or >26.5 μmol/L within 48 h of surgery compared with the preoperative value (1). Although such elevations are usually transient, they are associated with increases in morbidity and mortality (2, 3). The cause of these elevations is likely multifactorial and includes perioperative hypotension (4–6), high body mass index, restrictive fluid therapy (3, 7) and possibly oliguria (8, 9).

Serum creatinine is considered a lagging indicator of AKI that only increases when >50% of renal function is lost (10). Moreover, it is reported to be non-diagnostic in 48% of moderate-to-severe cases of AKI (11), and the values are also affected by muscle mass and hydration status (12). Importantly, serum creatinine often takes 24–48 h to rise in the context of AKI (13). Therefore, it is desirable to differentiate isolated elevations from those that reflect damage to kidney cells, which has a worse prognostic outcome.

A novel way of detecting AKI and determining the ‘stress’ of the kidneys involves analyzing the urine using the Nephrocheck® test (Astute Medical, San Diego, CA). This test calculates the AKIRisk™ index, which is a measure of the product of two cell-cycle arrest kidney injury biomarkers—insulin-like growth factor binding protein 7 (IGFBP7) and tissue inhibitor of metalloproteinase 2 (TIMP-2) (14–16). An uncorrected AKIRisk index >0.3 (mg/L)^2^ predicts moderate to severe AKI developing within 12 h, with a reported sensitivity of 92% and specificity of 46%.

The manufacturer of the Nephrocheck test states that AKIRisk values are the same regardless of whether correction for urine dilution is conducted. This is not logical, as correction for dilution needs to be applied to convert urine measurements from concentration to excreted amount/mass (17, 18). An overlooked fact is that the AKIRisk index should be corrected by the squared dilution because two variables are multiplied that need to be individually corrected for dilution (19).

The purpose of the present study was to examine how the components (i.e., IGFBP7 and TIMP-2) of the AKIRisk, alone and together after correction of the biomarkers TIMP-2 and IGFBP7 for urine dilution, compare with changes in serum creatinine after major surgery.

## Materials and Methods

### Permissions

This is a secondary report of a clinical trial of 72 adult patients (≥18 years of age) undergoing open pancreas, colorectal and liver resection surgeries between July 2018 and January 2019 at three university hospitals. The purpose of the primary study was to examine the effect of 16 mg IV dexamethasone and 100 ml 20% IV albumin solution on the serum concentration of glycocalyx degradation products (20). After Ethics Committee approval (number: 17/Austin/397), the trial was prospectively registered with the Australian New Zealand Clinical Trial Registry (ACTRN12618000361202). Written informed consent was obtained from all participants. Patient management was conducted according to usual local practice as previously described (20).

### Purpose and Patient Groups

The primary aim of this report was to assess IGFBP7 and TIMP-2 concentrations with and without correction for urine dilution. The secondary aims were to assess AKIRisk between the groups with and without correction for urine dilution. The correlations between the perioperative relative changes in serum creatinine and the corrected and uncorrected AKIRisk, IGFBP7, and TIMP-2 were also investigated. To differentiate between patients with high and low postoperative creatinine values, we selected data a priori from the 20% of patients who had the highest and lowest postoperative falls in serum creatinine values.

### Measurements

Blood for measurement of the serum creatinine concentration was collected immediately before the onset of general anesthesia in the morning of the operation day and at ~8 a.m. on the first postoperative morning. The analyses were performed at the certified hospital laboratory at the Austin Hospital, Melbourne, Australia.

A 5 ml midstream urine sample was aspirated from each patient's in-dwelling urine catheter at the same point in time and stored at −70°C until analyzed at Södertälje Hospital, Sweden. The frozen urine samples were thawed at room temperature immediately before analysis. The DCA Vantage™ analyser (Siemens Healthcare Diagnostics), a semi-automated, *in-vitro*, point-of-care benchtop platform, was used to measure microalbuminuria and the urinary creatinine concentration (measured by R.H.).

Another researcher (J.Z.) performed the Nephrocheck test on the same urine sample and at the same time on an Astute 140 meter (Astute Medical, San Diego, CA) after calibration with the Nephrocheck Liquid Control Kit. A fresh precision pipette was used for each transfer of urine to the test cuvette. The procedures given in the manual for the Nephrocheck test were followed.

We chose to compare the Nephrocheck results with serum creatinine on the first postoperative morning, implying that patients who suffered an injury to the kidneys during the surgery would show an increased release of biomarkers 12–24 h after surgery. Alternatively, patients who suffered an injury to the kidneys during the surgery would have a greater excretion of biomarkers than those who did not have kidney injury.

The following outcomes were measured: post/preoperative serum creatinine ratios, urinary creatinine concentrations, uncorrected Nephrocheck AKIRisk, dilution-corrected AKIRisk and albuminuria. Correction for dilution of the AKIRisk was obtained by dividing the values of the test components with the square of the urinary creatinine measured at the same time.

We included a separate reporting of the two components of the AKIRisk index, IGFBP7 and TIMP-2, which is normally unavailable to the users of Nephrocheck. These variables were reported with and without correction for dilution, the latter by dividing the values by the urinary creatinine concentration measured at the same time. Urine flow was measured during the first postoperative evening and night. The flow was expressed in ml/hour.

### Statistics

The data were reported as the median (25th−75th percentile limits) due to the frequent occurrence of skewed distributions. Selected comparisons were made using the Mann-Whitney and chi-square tests. A Wilcoxon matched pair test was used to compare measurements from different times. Correlations between variables were measured with simple linear regression where r = correlation coefficient. *P* < 0.05 was considered statistically significant. Given that this was an exploratory study, there was no correction for multiple testing.

## Results

### Serum Creatinine and Urine Flow

Fifteen patients with the greatest decrease and 15 with the greatest increase in serum creatinine in the perioperative period were selected to contrast the relationship between this variable and the AKIRisk index as measure by IGFBP7 and TIMP 2. Blood and urine were collected from all 30 patients. The median operating time was 4.9 (25th−75th percentiles, 3.9–8.4) h. The patient characteristics are summarized in Table 1.

**Table 1:** Demographic data and measurements of creatinine in the serum and urine before surgery and on the first morning after the surgery.

**Variable**	**Decrease in serum creatinine (*N* = 15)**	**Increase in serum creatinine (*N* = 15)**	**Mann-Whitney *P*-value**
Age (years)	53 (49–69)	68 (62–72)	<0.05
Gender (females/males)	4/11	11/4	<0.01
ASA class I/II/III	0/5/10	0/4/11	0.70
Body mass index (kg/m^2^)	27.2 (22.6–30.1)	28.2 (24.3–32.7)	0.42
Received dexamethasone (N)^*^	7	7	1.00
Operating time (h)	4.2 (3.2–5.9)	5.8 (4.5–9.4)	<0.03
**C-reactive protein (μg/L)**			
Before surgery After surgery	6 (1–29) 50 (24–90.0)	4 (2–11) 68 (24–11)	0.75 0.36
**Serum creatinine (μmol/L)**			
Before surgery Before/after ratio	71 (64–85) 0.81 (0.77–0.86)	74 (54–77) 1.57 (1.36–1.64)	0.65 Grouping
**Urine creatinine (mmol/L)**			
Before surgery After surgery	11.8 (4.1–13.7) 9.1 (7.1–11.6)	13.7 (6.2–17.8) 11.5 (7.4–15.3)	0.26 0.08
Urine flow (ml/min)	1.33 (0.83–1.67)	0.75 (0.56–0.94)	<0.04
Creatinine clearance (m/min)^**^			
Before surgery After surgery	147 (91–192) 182 (99–230)	109 (81–142) 63 (48–108)	0.52 <0.01

*Data are the median and 25th−75th percentiles*.

* *Randomized in the primary study (20)*.

** *Postoperative urine flow was measured, but the preoperative was estimated from urinary creatinine*.

Patients with a rise in serum creatinine were more often females, were older, and had a longer operating time. The median postoperative change in serum creatinine in the two groups was −19% and +57%, respectively. Eight of the 15 patients in the latter group fulfilled the criterion for Stage 1 AKI by having a rise in serum creatinine by >50%. Three additional patients fulfilled the criterion by having a rise in serum creatinine by >26.5 μmol/L. Postoperative urine flow was significantly higher in the patients with a decrease in serum creatinine (1.33 ml/min, 0.83–1.67) as compared to those who had an increase (0.75 ml/min, 0.56–0.94; P < 0.04).

### Primary Outcome-Correction of IGFBP7 and TIMP-2 for Urinary Dilution

**Figure 1:**
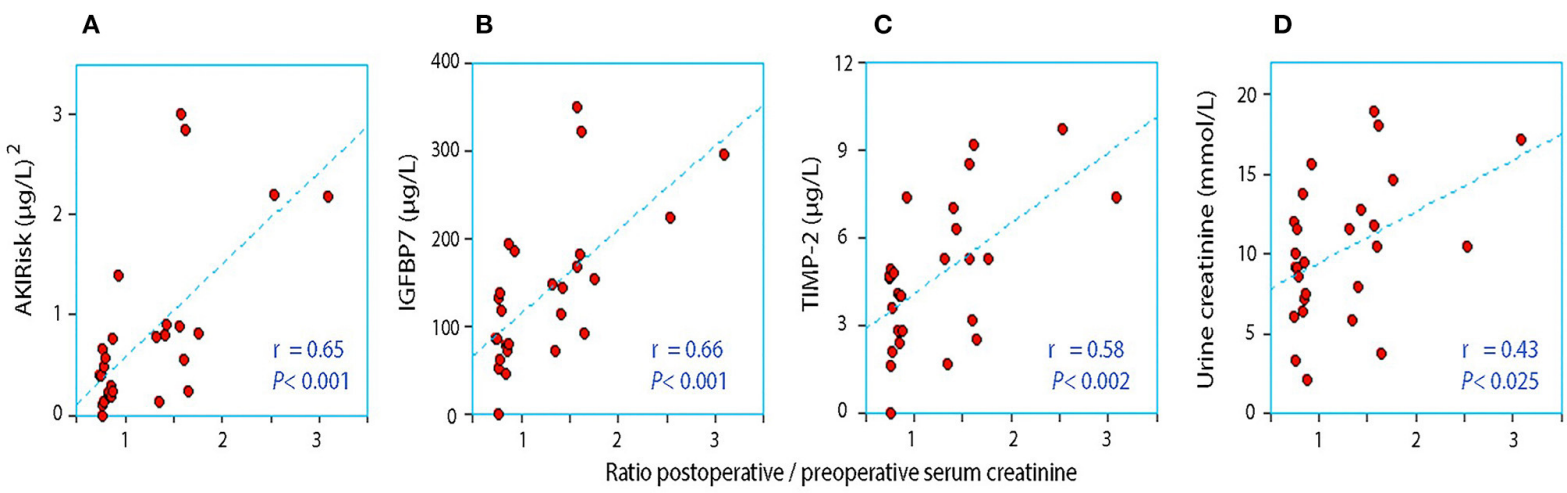
The relationship between the perioperative relative change in serum creatinine and the postoperative urinary concentration of **(A)** AKIRisk™, **(B)** IGFBP7, **(C)** TIMP-2, and **(D)** creatinine. No correction for sample dilution was conducted.

**Figure 2:**
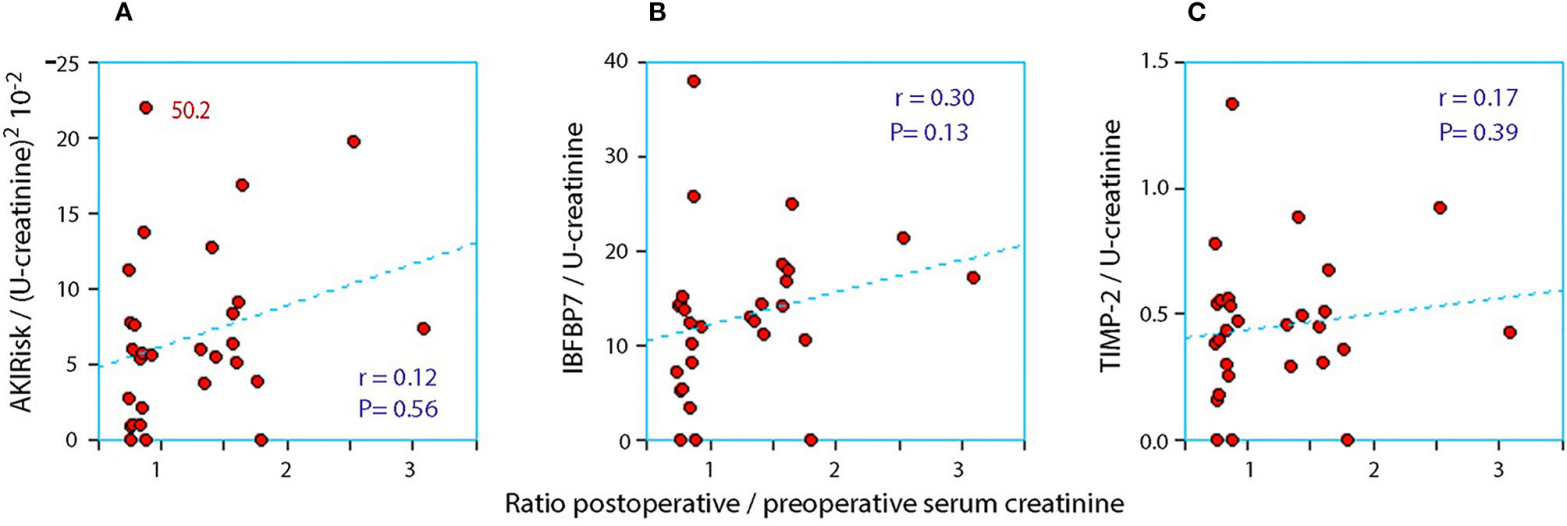
The relationship between the perioperative relative change in serum creatinine and the postoperative urinary concentration of **(A)** AKIRisk™, **(B)** IGFBP7, and **(C)** TIMP-2. The two biomarkers were corrected for sample dilution while the product, AKIRisk™, was corrected by the square of the sample dilution. An extreme outlier at 50.2 on the y-axis of in subplot SA has been moved down as indicated.

When the AKIRisk was corrected for urine dilution, there were no significant differences observed between the groups: 5.7 (2.1–7.8) μg^2^/mmol^2^ in patients with a decrease in serum creatinine, while being 6.9 (5.3–11.0) μg^2^/mmol^2^ in those with an increase in serum creatinine (*P* = 0.24). There was virtually no change in the latter group from before to after the surgery despite the marked increase in serum creatinine (Wilcoxon's matched pair test *P* = 0.69). A separate analysis of the two components of the AKIRisk index showed that IGFBP7 increased while TIMP2 decreased during the surgery (both *P* < 0.04), whereby their influence on the dilution-corrected AKIRisk index canceled out. On an individual patient level, the post/preoperative serum creatinine ratio correlated significantly with the output of the uncorrected Nephrocheck analysis (see Figure 1) but not when the output was corrected for urine dilution (see Figure 2).

### Corrected and Uncorrected AKIRisk Index

Patients had a high incidence of elevated AKIRisk index values both before and after the surgery. An uncorrected AKIRisk index >0.3 (mg/L)^2^, which predicts the risk of moderate to severe AKI, was recorded in 63% of the patients before the surgery and 65% on the first postoperative morning. The higher, often cited risk limit, >2.0 (mg/L)^2^, was reached by 23% of the patients before surgery and 13% during the follow-up. Measurements conducted with the Nephrocheck and DCA Vantage are presented in Table 2.

**Table 2:** Urine analyses of kidney injury biomarkers before surgery and on the first morning after the surgery.

**Variable**	**Decrease in serum creatinine (*N* = 15)**	**Increase in serum creatinine (*N* = 15)**	**Mann-Whitney *P*-value**
**No correction for dilution**		
**AKIRisk**™ **(mg/L)**^**2**^			
Before surgery After surgery	0.70 (0.09–1.98) 0.35 (0.19–0.57)	0.57 (0.22–1.53) 0.85 (0.67–2.20)	0.946 <0.0055
**IGFBP7 (μg/L)**			
Before surgery After surgery	130 (41–205) 83 (73–131)	119 (68–168) 161 (128–261)	0.93 <0.0064
**TIMP-2 (μg/L)**			
Before surgery After surgery	4.3 (2.6–12.4) 4.0 (2.8–4.7)	4.8 (3.3–8.6) 5.8 (4.3–8.0)	0.71 <0.02
**With correction for dilution**		
**AKIRisk**™ **(μg/mmol)**^**2**^			
Before surgery After surgery	8.3 (3.9–15.5) 5.7 (2.1–7.8)	8.0 (3.2–11.7) 6.9 (5.3–11)	0.44 0.24
**IGFBP7 (μg/mmol)**			
Before surgery After surgery	12.2 (8.5–17.0) 12.1 (7.3–14.4)	12.1 (8.5–14.6) 15.5 (12.7–18.3)	0.47 0.07
**TIMP-2 (μg/mmol)**			
Before surgery After surgery	0.65 (0.40–0.89) 0.45 (0.30–0.56)	0.62 (0.41–0.73) 0.46 (0.40–0.59)	0.65 0.68
**Albumin (mg/ml)**			
Before surgery After surgery	1.79 (0.84–5.01) 1.76 (1.14–3.21)	1.43 (1.10–3.27) 3.56 (1.45–12.36)	0.97 0.13

*Data are the median and 25th−75th percentiles*.

Patients with a perioperative decrease in serum creatinine showed lower Nephrocheck values on the first postoperative day than before surgery. By contrast, those with an elevation of serum creatinine had an increase in all Nephrocheck variables. The uncorrected AKIRisk decreased from 0.70 (0.09–1.98) to 0.35 (0.19–0.57) (mg/L)^2^ in patients with decreased serum creatinine. It rose from 0.57 (0.22–1.53) to 0.85 (0.67–2.20) (mg/L)^2^ in those who showed increased serum creatinine (group difference, *P* < 0.0055).

## Discussion

### Key Results

Our report investigated patients who underwent major abdominal surgery and demonstrated marked perioperative decreases and increases in serum creatinine values. The primary aim was to evaluate whether patients with marked elevated serum creatinine had reciprocal changes in IGFBP7 and TIMP-2 as assessed with the Nephrocheck. The results show that AKIRisk agreed reasonably well with the perioperative change in serum creatinine, while no correlation was found when the index was adequately corrected for urine dilution. After considering the amounts of excreted biomarkers rather than their concentrations, there was no difference in the AKIRisk between those with an average increase in serum creatinine by 57% compared to those with a median decrease of 19%. This implies that the AKIRisk index, up to the lower range of Stage 1 AKI, merely reflects the urine flow rather than an increased release of IGFBP7 and TIMP-2.

Urine becomes concentrated during surgery when the urine flow rate falls below 1.0 ml/min (21), which was the case in the group with an elevation of serum creatinine. Low flow may result in a reduced renal capacity for concentrating creatinine, resulting in incomplete elimination of creatinine load and accumulation of creatinine in serum postoperatively. Concentrated urine promotes the perioperative elevation of plasma creatinine even when found before the surgery (22), which receives some support from Figure 1D in our present study. Concentrated urine in daily life is primarily the result of low daily intake of water (23, 24). which is then a possible risk factor for the perioperative elevation of serum creatinine.

### Correction for Dilution

The manufacturer of Nephrocheck does not recommend correction for dilution, and, therefore, correction has not been routinely practiced by researchers who have published studies utilizing this method. The stunning deviation from the common practice of reporting results of urine measurements has been highlighted (17, 18, 24). The purpose of performing such a correction is to transform the concentration to amount/mass, which is logical as massive excretion of a biomarker can be masked by high urine flow.

Researchers who have utilized the Nephrocheck have argued that the AKIRisk distinguishes patients at risk of developing AKI, even if correction for dilution is conducted. However, we propose that AKIRisk should be corrected by the square of the dilution, because this index is the product of IGFBP7 and TIMP-2 that should separately be corrected for dilution.

Our findings suggest that the Nephrocheck's ability to distinguish between patients with markedly impaired ability to excrete creatinine after surgery is uncertain. The uncorrected AKIRisk appears to be a hybrid of biomarker excretion and urine flow. If an elevation of Nephrocheck persists after correction, it is plausible that a “renal injury” has occurred beyond urinary concentration. Comparing uncorrected AKIRisk with the corrected value is, therefore, clinically informative.

We acknowledge that certain substances should not be corrected for dilution, such as the glycocalyx degradation product syndecan-1, which is excreted at the same concentration regardless of urine flow (19). However, correction for dilution must be performed as long as the severity of the pathophysiological event is reflected by an increasing amount of biomarkers, which is likely the case with the biomarkers measured by the Nephrocheck.

### Literature

Nephrocheck data and serum creatinine were previously compared by Pajenda et al. (25). The authors found that AKIRisk increased before serum creatinine became elevated, which is in accordance with screening work conducted in our intensive care unit. This time delay indicates that at least 1 day is required for sufficient creatinine to accumulate in the serum to reach a detectable elevation. Cummings et al. found that Nephrocheck reliably indicated AKI after cardiac surgery (26).

Most studies with Nephrocheck focus on intensive care patients and, with few exceptions, have shown AKIRisk to be helpful in identifying patients at risk of developing renal failure (15, 27–29). However, in prior literature, the AKIRisk index has been used to detect injury to kidney cells, oliguria or both. In the present report, correcting for urine creatinine concentration showed that AKIRisk appeared to solely detect oliguria in the studied range of serum creatinine changes. Without adequate correction for sample dilution, the implications of the Nephrocheck test may become fundamentally different.

### Limitations

Limitations included the small size of the cohort, as well as that only 73% of patients (*n* = 12) in the high creatinine group reached the diagnostic criteria for AKI. Nephrocheck was originally intended for intensive care. However, the marked inflammatory response indicated by our measurements of C reactive protein showed that the surgeries of greater than 5-h duration had a physiological effect comparable to severe disease. AKI after surgery is well described (1–9), and the mechanisms that give rise to AKI are unlikely to be fundamentally different between intensive care and major surgery.

While the Nephrocheck may indicate imminent AKI in patients with undisputed renal cell damage, our findings suggest that perioperative elevation of serum creatinine should be adequately corrected for dilution before being used to indicate injury to kidney cells. Exceptions to this include patients with established kidney disease who have elevated baseline levels of serum creatinine (30).

The correction for dilution was made by dividing biomarker values with the urinary creatinine concentration, which might be confounded by AKI *per se*. However, all patients in the present study had normal renal function preoperatively. Any perioperative reduction of the creatinine excretion would increase the postoperative dilution-corrected AKIRisk index and not confound our main conclusion. Moreover, a recent study shows that the choice of biomarker for urine dilution (urine-specific weight, urine creatinine or urine osmolality) does not matter much in patients with the magnitude of serum creatinine changes reported here (19). The separate analysis of the two components of the AKIRisk index showed that the dilution-corrected IGFBP7 increased with serum creatinine, while TIMP-2 fell by almost the same percentage. The reason for the different reactions of the biomarkers is unclear.

Serum creatinine was also measured on the second postoperative day. Overall, elevated values were slightly lower than on the first postoperative day. The time-lapse between the supposed “kidneys stress” event was considered too long (36–48 h) to adequately capture the resulting increased release of biomarkers. Creatinine clearance was calculated based on the measurement of urine flow after surgery. The urine flow was not measured preoperatively and was estimated assuming an unchanged excretion of creatinine. We acknowledge the limitations of this approach.

## Conclusion

In the setting of major abdominal surgery, after correction of the biomarkers TIMP-2 and IGFBP7 for urine dilution, the Nephrocheck AKIRisk scores were significantly different from the uncorrected values. These finding imply that the AKIRisk index is a function of urine flow in addition to an increased release of the biomarkers.

## Data Availability Statement

The raw data supporting the conclusions of this article will be made available by the authors, without undue reservation.

## Ethics Statement

The studies involving human participants were reviewed and approved by Austin Health Human Research Ethics Committee. The patients/participants provided their written informed consent to participate in this study.

## Author Contributions

RH: study conception and design, data analysis and interpretation, statistical analyses, and writing of manuscript. FY: collection of specimens, data interpretation, and writing of manuscript. JZ: analyses of biomarkers, screening of literature, data interpretation, and writing of manuscript. ST: collection of specimens, screening of literature, and writing of manuscript. RB and LW: study conception and design, data interpretation, and writing of manuscript. All authors have read and approved the manuscript.

## Conflict of Interest

The authors declare that the research was conducted in the absence of any commercial or financial relationships that could be construed as a potential conflict of interest.

## Publisher's Note

All claims expressed in this article are solely those of the authors and do not necessarily represent those of their affiliated organizations, or those of the publisher, the editors and the reviewers. Any product that may be evaluated in this article, or claim that may be made by its manufacturer, is not guaranteed or endorsed by the publisher.
